# Movement protein of *Apple chlorotic leaf spot virus* is genetically unstable and negatively regulated by Ribonuclease E in *E. coli*

**DOI:** 10.1038/s41598-017-02375-y

**Published:** 2017-05-18

**Authors:** Rahul Mohan Singh, Dharam Singh, Vipin Hallan

**Affiliations:** 1 Plant virus lab, Biotechnology Division, CSIR- Institute of Himalayan Bioresource Technology, Palampur, (176061) H.P. India; 2 Academy of Scientific and Innovative Research (AcSIR), CSIR-Institute of Himalayan Bioresource Technology (CSIR-IHBT) Campus, Palampur, India; 3 Molecular and Microbial Genetics Lab, Biotechnology Division, CSIR-Institute of Himalayan Bioresource Technology, Palampur, (176061) H.P. India

## Abstract

Movement protein (MP) of *Apple chlorotic leaf spot virus* (ACLSV) belongs to “30 K” superfamily of proteins and members of this family are known to show a wide array of functions. In the present study this gene was found to be genetically unstable in *E. coli* when transformed DH5α cells were grown at 28 °C and 37 °C. However, genetic instability was not encountered at 20 °C. Heterologous over expression failed despite the use of different transcriptional promoters and translational fusion constructs. Total cell lysate when subjected to western blotting using anti-ACLSV MP antibodies, showed degradation/cleavage of the expressed full-length protein. This degradation pointed at severe proteolysis or instability of the corresponding mRNA. Predicted secondary structure analysis of the transcript revealed a potential cleavage site for an endoribonuclease (RNase E) of *E. coli*. The negating effect of RNase E on transcript stability and expression was confirmed by northern blotting and quantitative RT-PCR of the RNA extracted from RNase E temperature sensitive mutant (strain N3431). The five fold accumulation of transcripts at non-permissive temperature (43 °C) suggests the direct role of RNase E in regulating the expression of ACLSV MP in *E. coli*.

## Introduction

*Apple chlorotic leaf spot virus* (ACLSV), a member of genus *Trichovirus*, family *Betaflexiviridae*, is a positive sense, single stranded RNA virus having particle size of about 600–700 nm. The virus is known to infect apple, plum, cherry, peach, pear and apricot^1–3^. ACLSV along with *Apple stem pitting virus* (ASPV) and *Apple stem grooving virus* (ASGV) could result in 30% yield reduction in apple cultivar Golden Delicious^4^. ACLSV is one of the causative agents associated with lethal top working disease of apple trees in Japan, when Maruba Kaido (*Malus prunifolia var. ringo*) is used as a rootstock. The ACLSV genome encodes for three open reading frames (ORF’s) namely RNA dependent RNA polymerase (RdRp), Movement protein (MP) and a Coat protein (CP)^5, 6^. The movement proteins are usually required for movement of viruses^7^. Presently, MP’s are known to perform cell to cell movement by two different modes; (i) Type-1:MP forms a MP-gRNA complex and moves the viral genome to adjacent cells, the movement may or may not be dependent on other viral proteins e.g *Tobacco mosaic virus* and *Cucumber mosaic virus* ^8–10^, (ii) Type-2:virions are transported via tubules formed by MP inside the plasmodesmal pore^11–14^. Irrespective of the various modes of cell to cell movement, based on structural conservation, many viral movement proteins including the ACLSV MP are assigned to “30 K” superfamily (members contain a conserved D motif) of proteins^15, 16^.

Proteins belonging to the “30 K” superfamily of proteins are encoded by almost all groups of plant viruses, except viruses having double stranded RNA as their genome^17^. ACLSV MP is known to exhibit following properties: (i) localizes to cell wall^18^, (ii) forms protruding tubular structures at the cell membrane when expressed in protoplasts^18^; (iii) blocks systemic silencing^19^ and (iv) has two single stranded nucleic acid binding sites which are independently active of each other^20^. It is speculated that ACLSV MP transports the viral genome via MP-RNA complex (Type 1 transport), however, it is not yet clear why some Type 1 movement proteins (including ACLSV MP) show protruding tubular structures (Type 2 transport) in protoplasts^21, 22^. Although many functions of ACLSV MP are known but the underlying mechanisms behind most of these functions remain obscure. To understand the evolutionary origin, functional characteristics and the potential of “30 K” superfamily proteins to show interaction with other macromolecules; crystal structure of a “30 K” MP is required.

Theoretically, cloning of a DNA fragment seems to be an easy process. However, cloning of many genes and DNA fragments in certain insert:vector combinations pose painstaking problems because of the instability of certain DNA fragments. Although extensive work on instability of plasmids has been reported but still it is poorly understood. Plasmid instability may be caused by the following factors (i) copy number of plasmid, (ii) toxic gene products, (iii) metabolic burden on host cells to maintain the plasmids, (iv) recombination potential of certain inserts, (v) presence of cryptic bacterial promoters in eukaryotic sequences may produce spurious/toxic peptides which create problems in stabilization of plasmids or leads to promoter occlusion^23–25^, (vi) secondary structures of insert-﻿ DNA or presence of AT rich regions^26^, poly d(T) regions^27^ and presence of multiple repeats^28, 29^. While cloning a DNA fragment the instability may occur at initial colony formation stage, during subsequent regrowth in small scale liquid cultures or during large scale liquid cultures^30^. Despite having a huge knowledgebase on instability of plasmids in bacterial cells, it is nearly impossible to predict the stage at which a particular DNA segment would make the plasmid unstable.

Overcoming the instability posed by a sequence is a thorn in the foot, which can be taken care of by (i) growing cells at lower temperature^31^ and (ii) optimum choice of promoters and origin of replication. For inserts that have sequence constraints, different *E. coli* strains such as Stbl2™, Stbl4™^32^ and SURE^®^, that give increased stability to unstable plasmids can be used. Despite of availability of these problem solving methods, certain sequences may still be difficult to propagate in *E. coli*.

Proteins expressed heterologously/homologously in *E. coli* have been used for elucidating crystal structures of about 90% of the proteins submitted in Protein Data Bank (pdb)^33, 34^. Since the abundance of movement protein(s) in their natural environment would be too low to isolate enough protein(s) for undertaking functional and structural studies, therefore heterologous expression presents a formidable approach for structure elucidation. However, heterologous expression of various proteins in *E. coli is* marred by intrinsic problems such as instability of chimeric clones, toxicity caused by expressed proteins, improper folding, poor targeting and undesirable post transcriptional regulations. Stability of mRNA during heterologous expression is the foremost rate limiting factor and is of upmost importance^35–37^. The presence of sequence and structural constraints make the mRNA accessible to various endo and exoribonucleolytic enzymes. Amongst such enzymes, RNase E (endoribonuclease E) has a definitive role in the general chemical decay of bacterial mRNAs^38^. RNase E (conserved in proteobacteria) is essential and is a homo-tetramer of the protein (containing 1061 amino acids) encoded by *rne* gene of *E. coli*. It is important to note that the null mutants of *rne* are not viable^39^. The RNase E enzyme is well known for its functional roles in various RNAs that include rRNA and tRNA, non-coding RNAs and mRNA’s^40–43^. The presence of AU rich single-stranded loop in secondary structure of mRNA serves as a recognition site for RNase E^44–48^. RNAs with a 5′ monophosphate are cleaved preferentially by RNase E than the RNAs with 5′ triphosphates^49–51^. Recently, Clarke *et al*.^52^ reported a direct mode of entry by RNase E thus changing the previous paradigm of presence of 5′ monophosphate as the prime requisite for degradation and processing function of RNase E.

To elucidate the molecular mechanisms by which ACLSV MP counters the host defense, its interaction with host macromolecules and to have a crystal structure of this protein we intended to clone and express *ACLSV MP* heterologously in *E. coli*. In this itinerary, during cloning and heterologous expression of *ACLSV MP*, we encountered problems related to genetic instability and heterologous expression of *ACLSV MP* in *E. coli*. We attempted to decipher the molecular basis of expression hurdle using western blotting and transcript accumulation analysis in mutant strains of *E. coli*. Our data suggests that RNase E of the host *E. coli* negatively regulates the expression of ACLSV MP by cleaving *ACLSV MP* mRNA near the 3′-end.

## Results

### Cloning and instability of ACLSV MP gene

Based on nucleotide similarity the amplified *ACLSV MP* was found similar to ACLSV-P isolate. To study the structural and functional properties of ACLSV MP, we wish to clone and express *ACLSV MP* in various propagation and expression vectors. Therefore, in the present study we cloned *ACLSV MP* into pGEMT-Easy, pSMART, pET 32a (+), pET 28a (+), p5X and c5X vectors. However, in all attempts, cloning of full-length MP gene in pGEMT-Easy and pET vectors was found to be problematic and lethal to *E. coli* DH5α cells, when transformed cells were grown at 37 and 28 °C but not at 20 °C. At 28 °C and 37 °C none of the colonies that appeared after transformation had a correct full-length clone. It was found that either the positive colonies had a truncated fragment (~1000 nt) or had a non-sense mutation. In order to identify the problematic region in this gene, attempts were made to clone 3′-end (75 nt tandem deletions) and 5′-end deletion mutants in pGEMT-Easy vector. Results revealed that 375 nucleotides at 3′-end were causing problems in cloning when cells were propagated at 28 °C and 37 °C. However, when a low copy number vector pSMART LC-Amp (which has transcriptional terminators at both its ends) was used, it generated stable plasmids at 28 °C and 37 °C. Similar cloning problems were encountered when we tried to clone full-length MP gene in pET vectors. However, 3′-end truncated fragment was easily cloned in pET 32a+ and 28a+ vectors. Interestingly the *ACLSV MP* transcript has two start codons with a difference of two amino acids between the two AUG codons. Consistent with genetic instability results the new variants of plasmids generated with downstream ATG displayed similar results. This showed that the choice of AUG codon does not alter the stability of this gene in *E. coli* at 28 °C and 37 °C.

### Heterologous expression of full-length and 3′ truncated fragment of *ACLSV MP*

As stable cloning of full-length *ACLSV MP* in pET vectors was difficult, therefore for production of antibodies against ACLSV MP, 3′-end truncated fragment was cloned in pET 32a (+) and pET 28a (+) vectors. Overexpression of truncated fragment was found to be optimal only in pET 32a (+) vector at 30 °C. Purified overexpressed fusion protein was used for generation of anti-ACLSV MP antibodies in rabbits.

**Figure 1:**
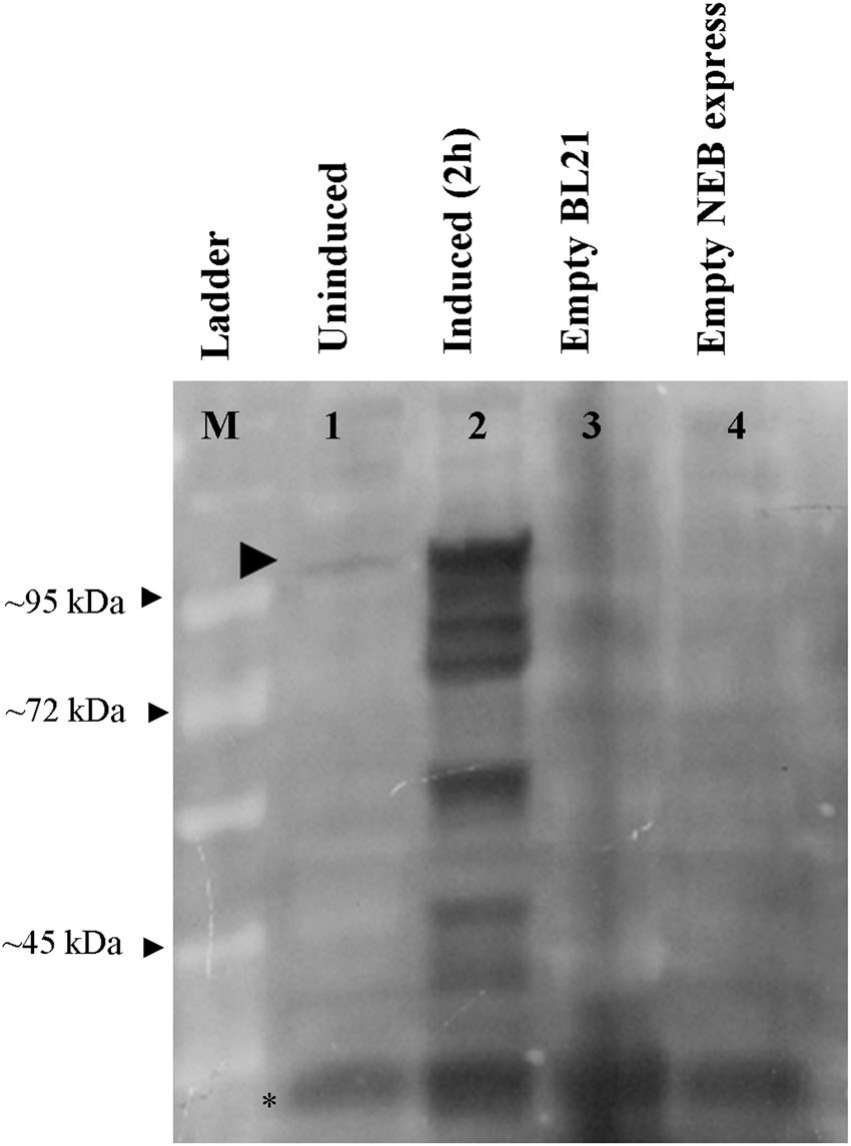
Western blot analysis done using lab raised antibodies, showing cleavage of full length fusion protein during induction. Lane M contains prestained protein ladder, Lane 1 contains uninduced cells, basal expression of full-length protein is marked with an arrow, Lane 3 contains total protein from 2 hr induced sample, full-length protein along with number of degraded products are visible. Lane 3 and 4 contains total proteins from wild type BL21 DE3 and NEB express cells. No cross reactivity with total proteins from wild type strains was observed. The low molecular bands observed (marked with*) at the bottom of western blot represent the non-specific bands observed in all lanes.

For heterologous expression of full-length *ACLSV MP*, the sequence was cloned in pMAL vectors (p5X and c5X) which contain bacterial *malE* gene under the control of *P*_*tac*_ promoter (utilizes *E. coli* RNA polymerase) and *malE* translation signals, which leads to robust protein expression and efficient solubilization of expressed protein. The constructs were induced at 16 °C, 28 °C and 37 °C for different time periods (see Table 1) with 0.3 mM IPTG and total protein was run on 12% SDS-PAGE. Gels were stained with coomassie brilliant blue (CBB) to visualize the expressed fusion protein. In coomassie stained gels fusion protein (~93 kDa) was not observed in any of the samples, whereas in control lanes an overexpressed maltose binding protein was clearly visible (Data not shown). However, western blot analysis using antibodies raised in the lab revealed multiple protein bands in IPTG induced (2 h induction) sample, whilst a slightly faint intact band of ~93 kDa was observed in uninduced sample (Fig. 1). The presence of cleavage products suggested that the expressed protein was unstable and probably had undergone proteolytic degradation. The antibodies were found to be specific and did not show any cross-reactivity to total proteins from wild type BL21 DE3 and NEB express cells. The result of western blotting clearly indicates that upon production of full-length ACLSV MP, this protein readily undergoes degradation/cleavage. The degradation of protein by the host machinery could probably be a mechanism employed by the host to save itself from toxic effects of ectopically expressed proteins.

**Table 1:** Different temperatures used for protein induction and sample collection time at respective temperatures.

Temperature	Sampling time		
16 °C	4 hrs	12 hrs	—
28 °C	½ hr	1 hr	6 hrs
37 °C	2 hrs	—	—

### Instability and toxicity caused by ACLSV MP during heterologous overexpression

**Figure 2:**
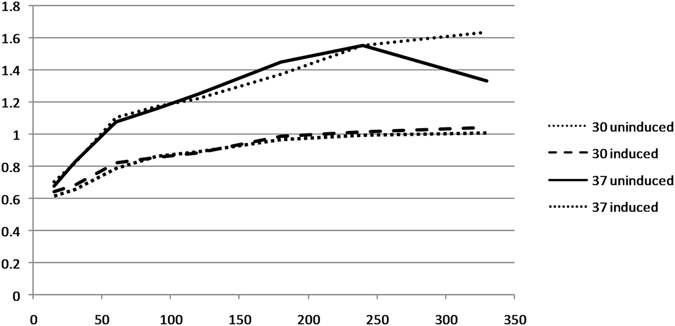
The graph has been plotted for O.D_600_ (Y-axis) and different time points (X-axis) of transformed NEB express cells grown with and without 0.3 mM IPTG at 30 °C and 37 °C. Solid straight line and round dotted line represents the growth of transformed cells without induction at 37 °C and 30 °C respectively. Broken solid black line (30 °C) and solid squares (37 °C) represents the growth pattern of transformed cells after induction.

Plasmid instability was evaluated by over-expressing the full-length *ACSLV MP* using pMAL vectors (for experimental procedure please sees materials and methods section). Out of total colonies that harbored the plasmid, 4.9 (*pc5X*/NEB express) and 13.8% (*pp5X*/NEB express) cells lost the plasmid during induction in case of vectors control. Whereas, the number of cells that lost plasmid in case of chimeric vectors rose to 70 (*pp5X*+/NEB express) and 21.8% (*pc5X*+/NEB express), respectively. Secondly, the number of cells that had mutated plasmids was found to be 8.7% and 17.15% for *pc5X*+/NEB express and *pp5X*+/NEB express, respectively. However, in case of vector controls none of the colony contained mutated plasmids. This data clearly shows that *ACSLV MP* is unstable in *E. coli* and further supports our gene instability results. Growth curve of NEB express cells producing fusion protein (mbp + ACLSV MP) decreased considerably throughout as compared to cells containing vector control. Upon overexpression the cellular energy is diverted towards translation machinery that significantly reduces the growth of cells. However, pMAL vectors (p5X and c5X) produce a 45 kDa maltose binding protein. Therefore, the comparison between growth statistics of cells producing maltose binding protein and chimeric fusion protein would give a relatively clear picture of toxicity caused by expression of heterologous protein. The decrease in growth (Fig. 2) of cells (*pc5X*+/NEB express) at 30 °C and 37 °C shows the toxicity imparted by ACLSV MP.

### Secondary structure prediction of ACLSV MP transcript

**Figure 3:**
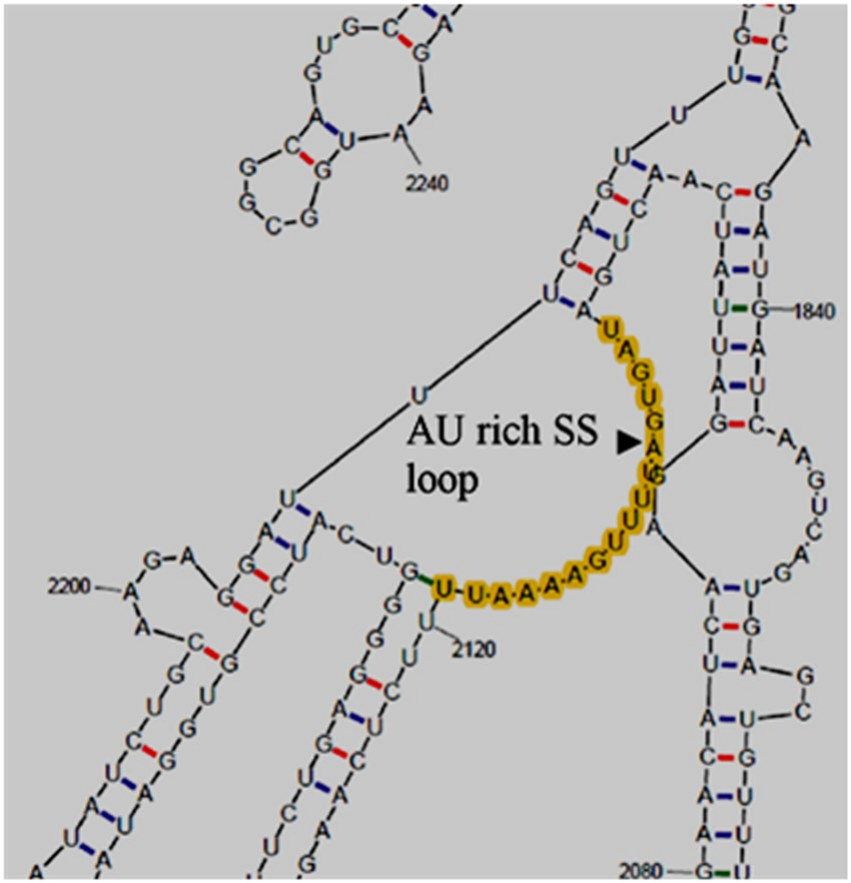
Part of the predicted secondary structure of chimeric (*malE* + *ACLSV MP*) RNA showing the single stranded (SS) AU rich loop spanning the region between nucleotides 2103–2119 (highlighted with yellow color) which is a potential site for cleavage by RNase E. The secondary structure was predicted at mFOLD web server (http://unafold.rna.albany.edu/?q=mfold/RNA-Folding-Form). This is a cropped image; full image is presented in supplementary Figure 1.

The presence of ribose sugar in RNA gives it an enhanced ability to form more hydrogen bonds as compared to DNA. As a result, RNA tends to form complicated secondary structures. RNA secondary structure and the conformational features govern the stability and final turnover of mRNA. In order to identify structural constraints which may affect the expression of ACLSV MP in *E. coli*, the secondary structure of fusion RNA (*malE* + *ACLSV MP*) was predicted using mFOLD web server^53^. The secondary structure revealed a characteristic “AU” rich single-stranded loop starting at position 2103 (nt) and ending at position 2119 (Fig. 3). AU-rich single stranded loops in mRNA secondary structure are potential targets for RNase E cleavages^54^. Likewise, when the secondary structure of 3′-end truncated ACLSVMP gene was predicted, no AU-rich single-stranded loop was observed. In order to confirm the role of RNase E in the degradation of *ACLSV MP* mRNA, we transformed *pc5X*+ in N3431 cells (temperature sensitive RNase E mutant, the strain is abbreviated as *rne* ^*ts*^ elsewhere in this article) and analyzed the *ACLSV MP* transcript profile using northern blotting.

### Transcript Profiling of ACLSV MP mRNA in RNase E mutant strain and *E. coli* DH5α

**Figure 4:**
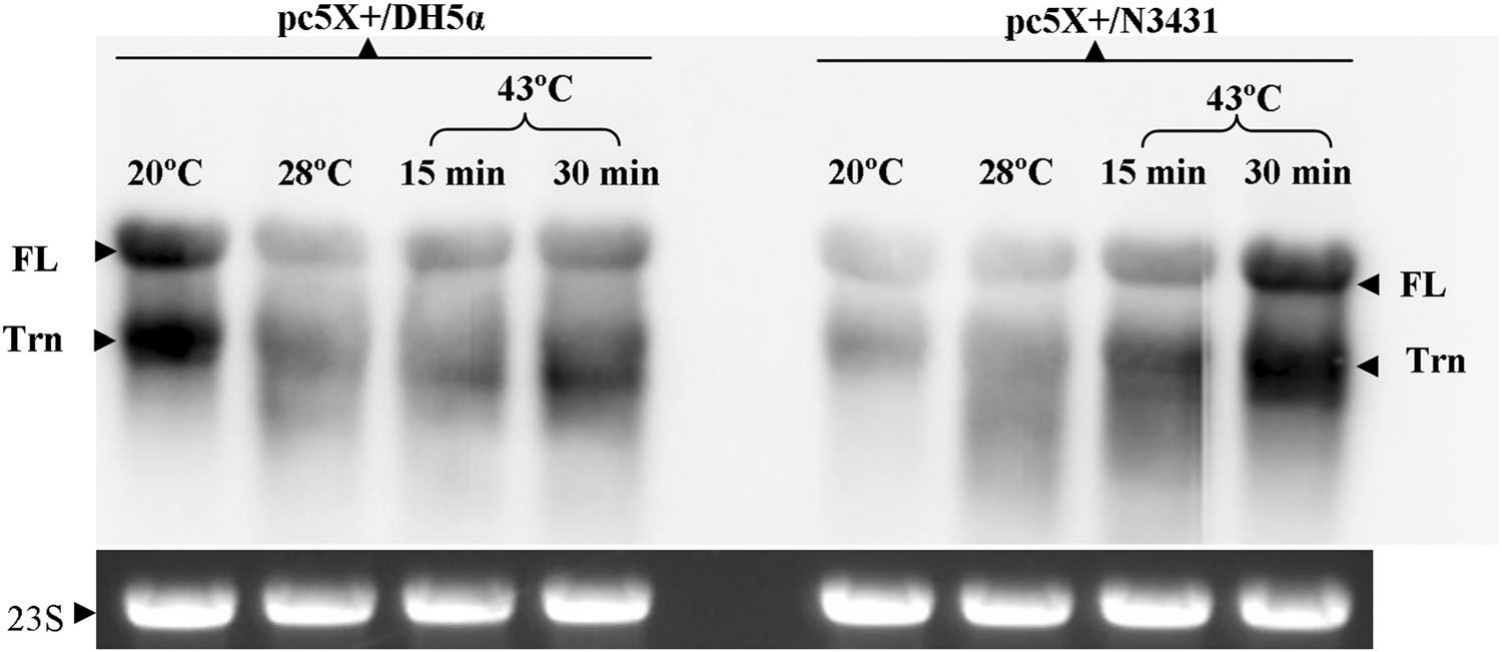
Northern blot image showing fate of chimeric *ACLSV MP* mRNA in *E. coli* DH5α (left panel) and in *rne* ^*ts*^ mutant (right panel). The chimeric plasmid *pc5X*+ was transformed into DH5α cells (*pc5X*+/DH5α) and into *rne* ^*ts*^ (*pc5X*+/N3431). Total RNA was used for northern blotting. The DH5α panel shows higher accumulation of both full-length (FL) and truncated (Trn) transcripts at 20 °C as compared to 28 and 43 °C samples. On the other hand, there is more accumulation of FL in sample collected after 30 minutes of shifting to non-permissive temperature (43 °C) compared to FL at 20 °C, 28 °C (permissive temperature) and 15 min sample of 43 °C (non-permissive temperature) in *pc5X*+/N3431 samples. EtBr stained 23 S ribosomal RNA (internal loading control) has been shown as a separate panel in the above image. The 23 S image is cropped from the total RNA image which is presented as a full length image in supplementary Figure 2.

The stability of mRNA is an integral and important aspect that could limit the level of overexpression in any heterologous system. Considering the probable cleavage of *ACLSV MP* transcript by RNase E, the *pc5X*+ was transformed into *E. coli* strains DH5α and *rne* ^*ts*^. Total RNA isolated from the above strains (DH5α and *rne* ^*ts*^) were subjected to northern blotting using a gene specific riboprobe. Results revealed that there was a significant accumulation of full-length fusion transcripts in *rne* ^*ts*^ strain at non-permissive temperature (43 °C), as compared to full-length transcripts present at 20 °C and at permissive temperature of 28 °C (Fig. 4). The accumulation of transcripts at non-permissive temperature clearly indicates the role of RNase E in stability of the chimeric mRNA. On the other hand, the transcripts from transformed DH5α cells under two different temperatures (i.e 28 °C and 43 °C) did not show alteration in accumulation of the full-length transcript. However, at 20 °C there was a significant accumulation of both full-length and truncated transcript as compared to other temperatures (28 °C and 43 °C), suggesting the role of temperature in genetic stability encountered at 20 °C in DH5α cells. It is also worth mentioning here that DH5α and *rne* ^*ts*^ are not isogenic strain, therefore, variable transcript profile at 20 °C in both cases is as expected. Relative quantification of *ACLSV MP* transcripts using real-time quantitative PCR showed 4.6 and 5.8 fold increase in corresponding transcripts at 15 and 30 min after shifting to non-permissive temperature (43 °C). This data is in harmony with the results obtained by northern blotting.

## Discussion

All of the studies that describe the function(s) of ACLSV MP have been done using MP sequence from ACLSV-A isolate^6^. However, studies on MP sequence from ACLSV-P isolate which shares only 79.9% homology to ACLSV-A MP sequence have not been undertaken to date. In addition, there is a lot of ambiguity related to the initiation codon marked in gene sequences of *ACLSV MP* submitted in nucleotide database maintained by NIH, Bethesda, Maryland, USA. A number of sequences have an upstream inframe AUG codon, which in majority of the cases falls under high kozak consensus sequence^55^ have been misreported. In pGEM^®^-T Easy vector the full-length clone was unstable at 28 °C and 37 °C however, we found the clone was stable at a lower temperature i.e 20 °C. Therefore, it is tempting to speculate that the instability is due to (i) a possible prokaryotic promoter activity present in the sequence or (ii) the toxicity of expressed protein. In a study on *Japanese encephalitis virus* infectious clone^23^ it was found that the prokaryotic promoter activity displayed by nucleotides 54–120 is responsible for its instability which was taken care of by culturing the bacteria at 25 °C. The lower temperature markedly reduces the promoter activity and stabilises the clone in *E. coli*. In the present study, the clones were stably propagated at 20 °C, suggesting a possible role of a prokaryotic promoter sequence present in the sequence of *ACLSV MP*. Prokaryotic promoters in ACLSV MP gene sequence were predicted using an online program available at http://www.fruitfly.org/seq_tools/promoter.html. With a minimum promoter score kept at 0.8, seven sequences were identified which may have promoter activity (data not shown). The function of such a promoter sequence may be hindered at lower temperature resulting in the stability of clones. Presence of tandem repeats and dinucleotide repeats affects the stability of plasmids in *E. coli* ^28, 29^. Presence of tandem repeats in *ACLSV MP* were analysed by an online tandem repeat finder program^56^ (https://tandem.bu.edu/trf/trf.html), no tandem repeats were found by this method. Furthermore, the toxicity and plasmid instability (checked using pMAL vector constructs) which leads to product heterogeneity during overexpression is in accordance to the results of a study^57^, that showed cloning related problem of *S. pneumoniae mal*X and *ami*A genes in *E. coli* are not solely dependent on the strong promoters on multicopy plasmids but rather it is a significant effect of the gene products.

Full-length infectious clones of many single-stranded RNA viruses have been difficult to propagate in *E. coli* ^58^. Full-length ACLSV infectious clone was easily assembled by homologous recombination in yeast^58^. However, when the plasmids from yeast were transformed in *E. coli* and the transcripts generated from *E. coli* plasmids were checked for infectivity on host plants, the recorded infectivity was only 1–2%. Reasons behind such a low infectivity remain unknown. ACLSV MP is integral for movement of virus in host cells, it also functions as a suppressor of gene silencing and based on our instability results, it may be speculated that the low infectivity might have been due to incorporation of some non-sense mutations in MP sequence.

*In-vivo* deficiency of RNase E is known to prolong the half-life of ~1500 mRNAs^59^. In the present study we found the presence of an AU-rich single-stranded loop in RNA secondary structure and its negating effect on mRNA turnover [which was experimentally proved by increase in mRNA of chimeric construct in case of RNase E mutants (*rne* ^*ts*^)], is in accordance to many previous reports that investigated the role of RNase E in processing or turnover or both in case of many RNAs^42, 60^. The increase in relative abundance of *ACLSV MP* mRNA at non-permissive temperature (as evident from northern blot and qPCR data) confirms the negating effect of endonucleolytic enzyme RNase E on heterologous expression of *ACSLV MP*.

Heterologous overexpression of truncated *ACLSV MP* in *E. coli*, yielded protein in inclusion bodies that was seen as a distinct band (~60 kDa) in coomassie stained gels. On the other hand, during overexpression of full-length gene in pMAL vectors we did not see any fusion product (in coomassie stained gels). As transcription and translation are coupled in prokaryotes therefore inspite of the action of RNase E there would be certain transcripts that would miss ribonuclease action to make full-length protein. The western blot analysis showed presence of full-length fusion protein in addition to other smaller sized bands which could be due to the cleavage/degradation of full-length protein. These results infer that although there is very less expression of full-length fusion protein, but it is still prone to proteolysis. However, due to aggregation the protein from truncated fragment is saved from proteolysis, as inclusion bodies are resistant to proteolytic degradation^61^. The indigenously raised antibodies were highly sensitive and did not show any cross-reactivity to the total proteins from wild type BL21 and NEB expressed cells (Fig. 1).

Chimeric/tagged proteins are widely used in protein research for purposes as diverse as vaccine development, immunodetection, trafficking studies, biochemical purification and interaction studies^62^. Commonly used fusion tags include 6X histidine, MBP-maltose binding protein, GST- glutathione S-transferase, TRX-thioredoxin and Strep- streptavidin/avidin^63–67^. The formation of inclusion bodies due to lack of sophisticated pathways for post-translational modifications, is by far a major limiting factor in *E. coli* expression system^68–71^. Folding and solubility limitations (which lead to aggregation of expressed proteins) could be improved by fusing the protein of interest to expression tags^72^. It has been reported that MBP greatly enhanced the solubility of five out of six (otherwise expressed in insoluble form) different and diverse passenger proteins, whereas, the use of TRX or GST did not significantly alter the solubility of those proteins. Based on our results of truncated gene expression (protein expressed in inclusion bodies) and bioinformatic calculations for solubility index (0.695) for full-length gene, we predicted that the full-length gene would also form inclusion bodies that would be highly undesirable for structural studies, though the formation of inclusion bodies do have its own advantages as they can be easily isolated in relatively pure form and are resistant to proteolysis^73^. pMAL vectors, apart from increasing solubility also offers to export the protein to periplasm that is a more oxidizing compartment than cytoplasm and also hosts specific chaperons, foldases and enzymes for disulphide bond formation^74–76^. Thus, we tried to express the gene in both the cytoplasmic and periplasmic vector and found that proteolysis is occurring in both the cases (data not shown), which implies that the expressed protein is prone to proteolysis irrespective of the site of protein production.

In conclusion, the findings from present study have clearly shown that the cloning related problems of the movement protein of ACLSV can be taken care of by culturing at a low temperature (20 °C) but still post-transcriptional and post-translational controls pose a limitation to successful heterologous expression of *ACLSV MP* in *E. coli*. Although the present dataset is not sufficient to devise a proper strategy for overexpression of this gene in *E. coli*, it is necessary to study the differential expression of host genes during overexpression of the heterologous gene. The information generated from such studies would lead to better understanding of the underlying barriers that pose bottlenecks for overexpression of foreign genes. Once the conundrum of overexpression is solved, further structural studies would lead to a better understanding of the ACLSV MP function.

## Materials and Methods

### Cloning of ACLSV MP Gene and truncation of it’s 5′ and 3′-ends

**Figure 5:**
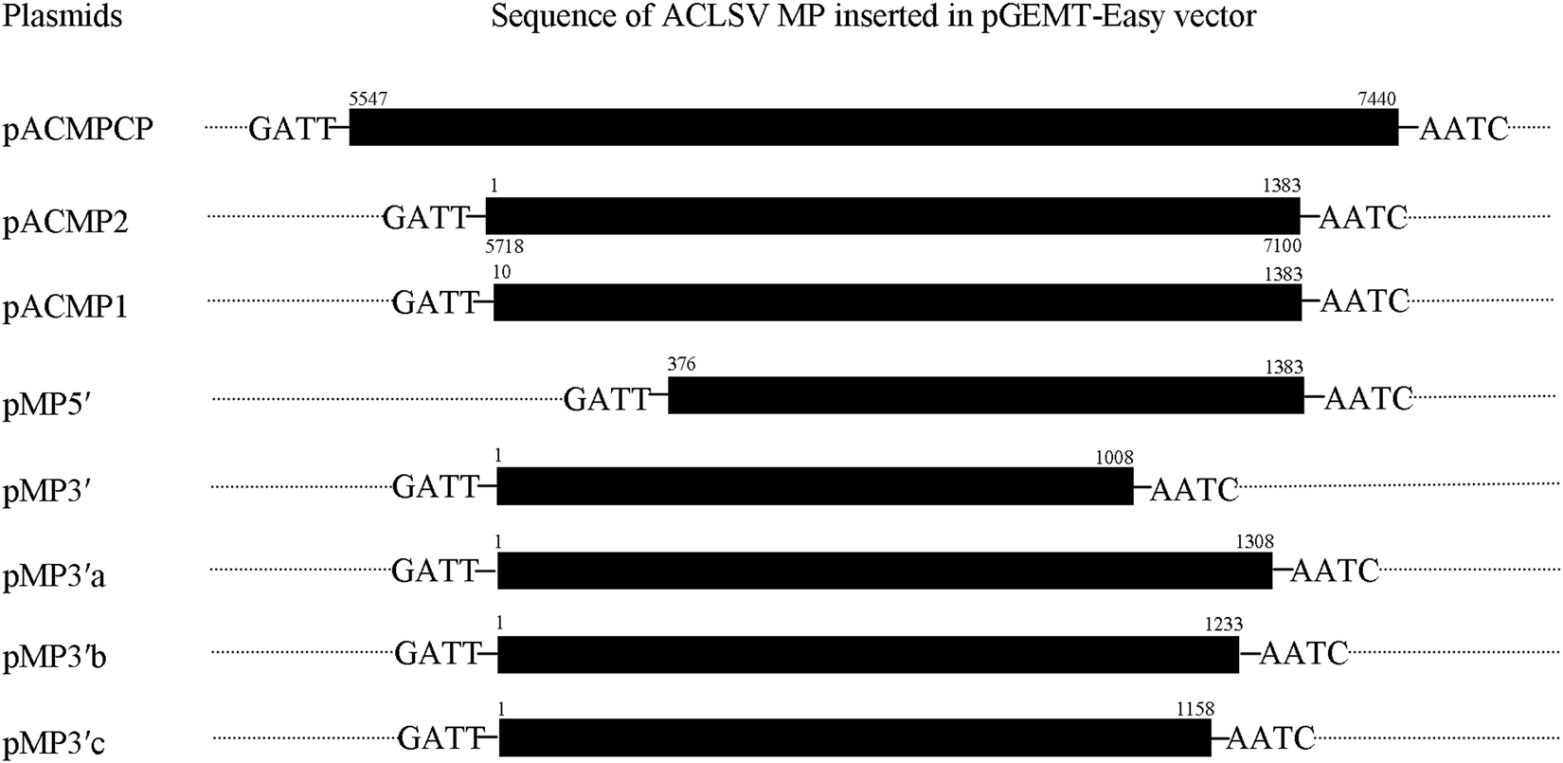
Different sequences of ACLSV genome used to clone *ACLSV MP* and different truncated mutants of *ACLSV MP*. Solid black rectangles represent the *ACLSV* sequences inserted in pGEMT-Easy vector. GATT, AATC and dots represent the vector sequences. *pACMPCP* contains genomic sequence of ACLSV spanning from 5547–7440 nt’s which contains full-length *ACLSV MP* and partial CP. *pACMP2* is full-length *ACLSV MP* gene (1383 nt’s), *pACMP1* contains *ACLSV MP* devoid of first nine nucleotides. *pMP5*′ is 5′-end truncated construct which lacks first 375 nt’s, *pMP3*′ lacks 375 nt’s from the 3′-end. *pMP3*′*a*, *pMP3*′*b* and *pMP3*′*c* contain 1–1308 nt’s, 1–1233 nt’s and 1–1158 nt’s of *ACLSV MP*.

For cloning *ACLSV MP* a ~2 kb region from ACLSV genome was amplified from virus infected apple cultivar Gala using primers AC5547 F and AC R 7440 (Table 2), which contains both MP and partial coat protein (CP) regions. The recombinant plasmid *pACMPCP* was generated by ligating the amplicon in pGEM^®^-T Easy vector and then transformed into *E. coli* DH5α cells. From the cloned sequence, *ACLSV MP* (1383 nt) was amplified using primers RMS125F and RMS125R. We attempted to clone this amplicon in pGEM^®^-T Easy vector, however this recombinant plasmid (*pACMP1*) was found to be unstable at 37 °C and 28 °C. The amplified *ACLSV MP* sequence was then ligated into a low copy number plasmid pSMART-LC Amp (Lucigen Corporation, Middleton, WI, USA) vector (which contains transcription terminators at both its open ends); to generate a recombinant plasmid named *pSACMP*. All the PCR amplifications for cloning purpose in this study were carried out using Advantage^®^2 polymerase mix (Takara Bio, USA). As the plasmid *pACMP1* was found to be unstable, so to identify the region in *ACLSV MP* that may be causing instability of MP sequence in DH5α cells, we generated following deletion mutants; *pMP5*′, *pMP3*′, *pMP3*′*a*, *pMP3*′*b* and *pMP3*′*c* (Fig. 5). The details of the primers used to generate the full-length and truncated clones are given in Table 2. Further to evaluate the role (in instability) of an inframe ATG that lie six nucleotides downstream of the first ATG in *ACLSV MP* sequence, we generated various chimeric plasmids like other plasmids generated in this study. All the full-length and truncated constructs were transformed into competent *E. coli* DH5α cells. Equal number of cells from each transformation were spread on three Luria agar plates supplemented with ampicillin (100 µg/ml), and one of each plate was kept at 20 °C, 28 °C and 37 °C. Each transformation was repeated thrice and checked for colony formation.

**Table 2:** Various primer combinations used for cloning of full-length, truncated and expression constructs of *ACLSV MP*.

S.No	Amplicon for plasmid	Primer pair used (sequence 5′ - 3′)
1	*pACMPCP*	**ACR5547F** - TCTGGAGTTTTCATTCGCCTA
		**ACR 7440R -** TGGGTTCAAGAGTTTATTTGCGTAAACATGC
2	*pACMP2*	**RMS125F –** *ATG*GCGACGATGATAAGGGGTCACAAATGA
		**RMS 125R -** TCACAAACCTGACGGAAGGTCATG
3	*pACMP1*	**RMS124F** – *ATG*ATAAGGGGTCACAAATGA
		**RMS 125R -** TCACAAACCTGACGGAAGGTCATG
4	*pMP5*′	**RMS ACF 1000-**AAGAGCAAGCAATGTTGCAGAAATT
		**RMS 125R–**TCACAAACCTGACGGAAGGTCATG
5	*pMP3*′	**RMS125F–** *ATG*GCGACGATGATAAGGGGTCACAAATGA
		**RMS AC Rev 1000 -** GNAGAHATGTTATCKACGGATGAC
6	*pMP3*′*a*	**RMS125F–** *ATG*GCGACGATGATAAGGGGTCACAAATGA
		**RMS Rev 752 -** ACCACCTCCTTCAGATTGAAGGA
7	*pMP3*′*b*	**RMS125F–** *ATG*GCGACGATGATAAGGGGTCACAAATGA
		**RMS Rev 753** - AACTCCGTCTGCTCCGAGGTTCC
8	*pMP3*′*c*	**RMS125F–** *ATG*GCGACGATGATAAGGGGTCACAAATGA
		**RMS Rev 754 -** CCTGTCTTTCCATGAAGGGGGCT
9	*pc5X*+, *pp5X+*	**RMS124FE-** CCATGGCGACGATGATAAGGGGTCACAAATGA
		**RMS 125RE -** GAATTCTCACAAACCTGACGGAAGGTCATG

The initiation codons in plasmids *pACMP1* and *pACMP2* have been italicized. For preparation of constructs for pMAL vectors the enzyme sites in primers RMS 124FE and RMS 125RE have been underlined.

### Secondary structure prediction of ACLSV MP

Amplified *ACLSV MP* sequence was used for the prediction of secondary structure of mRNA using mFOLD web server^53^. Similarly, secondary structures of truncated fragments 920 nt (5′-end) and 454 nt (3′-end) were also predicted using energy minimization program of mFOLD web server.

### Heterologous expression of ACLSV MP in *E. coli*

For heterologous overexpression in *E. coli*, *ACLSV MP* sequence was amplified using primers RMS 124FE (*Nco*I restriction enzyme site) and RMS125RE (*Eco*RI restriction enzyme site), after restriction digestion (with *Eco*RI and *Nco*I) and purification of digested PCR product it was ligated into similarly digested pMAL expression vectors (p5X and c5X) to generate recombinant plasmids *pc5X*+ and *pp5X*+. Recombinant plasmids were transformed into competent NEB express cells. For protein overexpression the cells (NEB express cells containing *pp5X*+, *pc5X*+ and vector controls) were grown in Luria broth amended with 1% glucose. After the cells reached an O.D_600_ = 0.5, IPTG (0.3 mM final concentration) was added. The cells were grown at three different temperatures (16 °C, 28 °C, 37 °C) and samples (1 ml culture) were drawn at different time points (Table 1). The samples were centrifuged to collect the cells. Equal cell mass was suspended in SDS loading dye (Laemelli loading buffer) and heated for 7 minutes on a boiling water bath. The samples were cooled on ice for 5 minutes, and analyzed on 12% SDS-PAGE, to see protein profile the gels were stained with CBB.

Truncated fragment (920 nt) was cloned in pET 32a+ and pET 28a+ vectors. Recombinant plasmids were transformed into BL21 DE3 cells, and a single positive clone (of each plasmid) was selected for protein overexpression. Induction was carried out at 30 ° C with 1 mM IPTG and equal number of cells were collected at different time intervals (2, 3 and 4 hr). Equal numbers of cells from each sample were then suspended in 500 µl phosphate buffer saline pH 7.0 containing 1 mM PMSF and were subjected to sonication. Cell pellets and supernatant were collected separately after centrifugation. Protein profile of both fractions (pellets and supernatant) was analyzed on 12% SDS-PAGE as mentioned earlier.

### Protein purification and antisera generation

Protein from truncated fragment (920 nt) cloned in pET 32a+ vector was used for generation of antisera against ACLSV MP. For recombinant protein production, one litre culture (2x YT media) was induced at 30 °C with 1 mM IPTG for 4 hrs, the cells were pelleted down and subjected to sonication for cell lysis. After sonication, the pellets were dissolved in 300 mM NaCl and 8 M urea, so as to denature the protein entrapped in inclusion bodies. Protein purification was carried out using Ni-NTA resin (Thermo scientific) as per manufacturer’s instructions and eluted in a buffer containing 300 mM NaCl, 8 M urea and 250 mM imidazole (elution buffer). Further, the eluted protein was dialyzed against a buffer system containing 100 mM Tris-HCL (pH-7.8) + 0.14 M β-mercaptoethanol and 2 M Urea. The purified protein was then used as an antigen for immunization in rabbits. Polyclonal antisera were raised by applying three weekly consecutive injections of 500 mg purified protein emulsified with complete Freund’s adjuvant (in an equal volume ratio) and injected subcutaneously and intramuscularly at five different sites into healthy white New Zealander male rabbits (approximately four months old). For the booster doses, incomplete Freund’s adjuvant was used. After 6 weeks, the animal was bled from the marginal ear vein and serum was collected from the blood using pasture pipette and centrifuged at 5000 rpm for 10 min at 4 °C. IgG was purified using IgG extraction kit (Bangalore Genei, Merck Biosciences, Germany) as per manufacturer’s instructions. For raising antisera, rabbits were used after approval from the Institutional Animal Ethics Committee at this institute (CSIR-Institute of Himalayan Bioresource Technology) which works under the guidelines of “Committee for the purpose of control and supervision on experiment on animals (CPCSEA)”. All the work done on animals in this study was approved by institutional committee on animal ethics, and experiments were executed as per the guidelines laid by the committee.

### Plasmid instability test and growth curve of transformed NEB express cells

The constructs prepared in pMAL vectors were subjected to plasmid stability test as described in pET system manual, 8^th^ edition, 1999 (Novagen, USA) with few modifications. Briefly, immediately before induction (O.D_600_ = 0.5) equal number of cells from empty vector containing NEB express cells and recombinant vector containing cells were spread on each of the following plates (i) Luria agar, number of colonies on this plate will represent the number of all viable cells (ii) Luria agar + ampicillin (100 µg/ml), colonies on this plate will represent the cells which carry the plasmid (iii) Luria agar + 0.3 mM IPTG, colonies on this plate will represent the plasmids/mutants that have lost the ability to express the target gene (iv) Luria agar + ampicillin (100 µg/ml) + IPTG (0.3 mM), colonies on this plate represent only the mutants that retain the plasmid but have lost the ability to express the target gene. The experiment was repeated thrice and colonies on each plate were counted. For graphical representation (Supplementary Fig. 3) average of the colonies from three independent replicates were taken. For study of growth kinetics of the transformed NEB express cells, vector controls (*p5X* and *c5X*) containing cells and chimeric plasmids (*pp5X* + and *pc5X*+) containing cells were propagated in Luria broth (50 ml) containing 100 µg/ml ampicillin and 1% glucose till the OD_600_ reached 0.5. After this 0.3 mM IPTG was added to each culture and then incubated at 30 °C and 37 °C. Absorbance at 600 nm was taken using spectrophotometer (Specord 200, Analytic Jena, Germany) at following time points: 15, 30, 60, 90, 120, 180, 240 and 330 min. The readings from this experiment were plotted in a graph to see the difference in growth kinetics of empty and chimeric vectors.

### Strains of *E. coli*

**DH5α-**F^−^ *endA1 glnV44 thi-1 recA1 relA1 gyrA96 deoR nupG purB20* φ80d*lacZ*ΔM15 (*lacZYA-argF*) U169, hsdR17(*r*_*K*_^−^ *m*_*K*_^+^), λ^−^. **BL21**-F^−^ *ompT gal dcm lon hsdS*_*B*_(*r*_*B*_^−^ *m*_*B*_^−^)λ(DE3[*lacI lacUV5*-*T7gene 1 ind1 sam7 nin5*]) [*malB* ^+^]_K-12_(λ^S^). **NEB express -** *fhuA2 [lon] ompT gal sulA11 R(mcr-73::miniTn10–TetS)2 [dcm] R(zgb-210::Tn10–TetS) endA1* Δ*(mcrC-mrr)114::IS10*. **N3431-** *lacZ43, relAI, spoTI, thi-1, rne-3071*(Goldblum and Apirion 1981).

### Transcript profiling

The transformed *E. coli* DH5α and *rne* ^*ts*^ were grown at 28 °C (3 cultures of each transformed cell type) and one batch each of both transformed cells was grown at 20 °C. After the cells reached O.D_600_ = 0.5, 0.3 mM IPTG was added for induction. After addition of IPTG, transformed *E. coli* DH5α and *rne* ^ts^ were grown at 20 °C and 28 °C for 1 hr and samples were collected (~1 × 10^9^ cells). Similarly, samples (15 min and 30 min) were collected from cultures post shift to non-permissive temperature (43 °C). At non-permissive temperature samples were collected at 15 min and 30 min. For transcript profiling, the total RNA from different IPTG induced samples [*E. coli* DH5α and N3431 temperature sensitive mutant (*rne* ^*ts*^) cells] were isolated using hot SDS-Phenol method^77^, DNAse treatment and subsequent purification of total RNA was done using a commercial kit (NucleoSpin^®^, Macherey Nagel, Germany). The presence of *ACLSV MP* specific transcript was detected by northern blotting. Equal amount (1.5 µg) of RNA from different temperatures and time intervals were loaded on formaldehyde denaturing agarose gel and then transferred to nitrocellulose membrane by capillary action. Anti-sense DIG labeled riboprobe prepared from 3′ -end truncated *ACLSV MP* was used for hybridization (at 68 °C). Chemiluminescent detection was carried out using CDP star (Ambion, Life technologies, USA) as per the manufacturer’s instructions. Images were captured using azure c300 imaging system (Azure Biosystems, USA).

### Real Time PCR

First strand cDNA synthesis was done with 1 µg of total RNA (DNase treated) isolated from permissive (28 °C) and non-permissive temperature (43 °C) conditions using Verso cDNA Synthesis Kit (Thermo Scientific, USA) in a 20 µl reaction containing 4 µl of 5X cDNA synthesis buffer, 500 µM each dNTP, 400 ng of random hexamer primer, 1 µl RT enhancer and 1 µl Verso enzyme mix. The reaction was incubated for 50 min at 42 °C, for inactivation of RT enhancer the reaction was then heated to 95 °C for 2 minutes. Relative quantification of *ACLSV MP* transcripts was done on Agilent Mx3000 P QPCR system using DyNAmo ColorFlash SYBR Green qPCR kit (Thermo scientific, USA). Calculations for relative abundance of *ACLSV MP* mRNA were done using *E. coli IdnT* as a reference gene. List of primers used in qPCR are listed in Table 3. For relative quantification of transcripts 2 µl of the 1:10 diluted cDNA and 2 µM of each forward and reverse primer with three technical replicates was used. Relative quantification was done by comparative ΔΔCT method^78^.

**Table 3:** List of primers used for qRT-PCR.

Gene	Primers (sequence 5′-3′)	Reference
**IdnT**	**EC idn T F-** CTGTTTAGCGAAGAGGAGATGC	79
	**EC idn T R-** ACAAACGGCGGCGATAGC	
**RNase E**	**EC rne F-** AGGCAAACATCTACAAAGGTAAAATC	This study
	**EC rne R-** AACACATCTTTAATGTTGGGACGAC	
**mal E**	**375 F–** AAGTGGATAACATATCTGCAAGAG	This study
	**375 R-** TCACAAACCTGACGGAAGGTC	

For quantification of transcripts of *ACLSV MP*, primers were designed from the nucleotide sequence of maltose binding protein present in pMAL vectors. The RNase E primers were designed from RNase E sequence derived from the genome of an *E. coli* K-12 strain MG1655 (GI:556503834).

### Western blotting

NEB express cells transformed with *pp5X*+ and *pc5X*+ were propagated and harvested as described earlier. Equal cell mass from cultures harbouring control and chimeric plasmids were resuspended in SDS loading dye (Laemelli loading buffer) and heated at 95 °C for 7 minutes and cooled on ice for 5 minutes. These samples were separated on 12% SDS-PAGE, transferred to PVDF membrane and blocked with 5% skimmed milk-Tris buffered saline amended with 0.1% Tween-20 (TBS-T) overnight at 4 °C. After blocking the membrane was washed five times with TBS-T and then incubated for 1 hr with anti-MP antibodies (lab raised) at room temperature (RT) with continuous slow shaking. Membrane was again washed several times (five times) with TBS-T and further incubated with 1:5000 anti-rabbit IgG conjugated to horseradish peroxidase (Sigma-Aldrich, USA) for 30 minutes at RT. Signals were detected using SuperSignal™ West Femto Maximum Sensitivity Substrate (Thermo Scientific, USA).

### Ethical approval and informed consent

For raising antisera, rabbits were used after approval from the Institutional Animal Ethics Committee under Committee for the purpose of control and supervision on experiment on animals (CPCSEA).

## Electronic supplementary material

Dataset 1
