# Supplementary material for: Movement protein of Apple chlorotic leaf spot virus is genetically unstable and negatively regulated by Ribonuclease E in E. coli
Source: Sci Rep. 2017 May 18;7:2133. doi: 10.1038/s41598-017-02375-y (PMC5437062; doi:10.1038/s41598-017-02375-y)
Supplement: Supplementary file 1 — Dataset 1 [file 41598_2017_2375_MOESM1_ESM.doc]

**Movement protein of *Apple chlorotic leaf spot virus* is genetically unstable and negatively regulated by Ribonuclease E in *E. coli***

Rahul Mohan Singh^1, 2^, Dharam Singh^♦,2,*^ and Vipin Hallan^1,2,*^

**
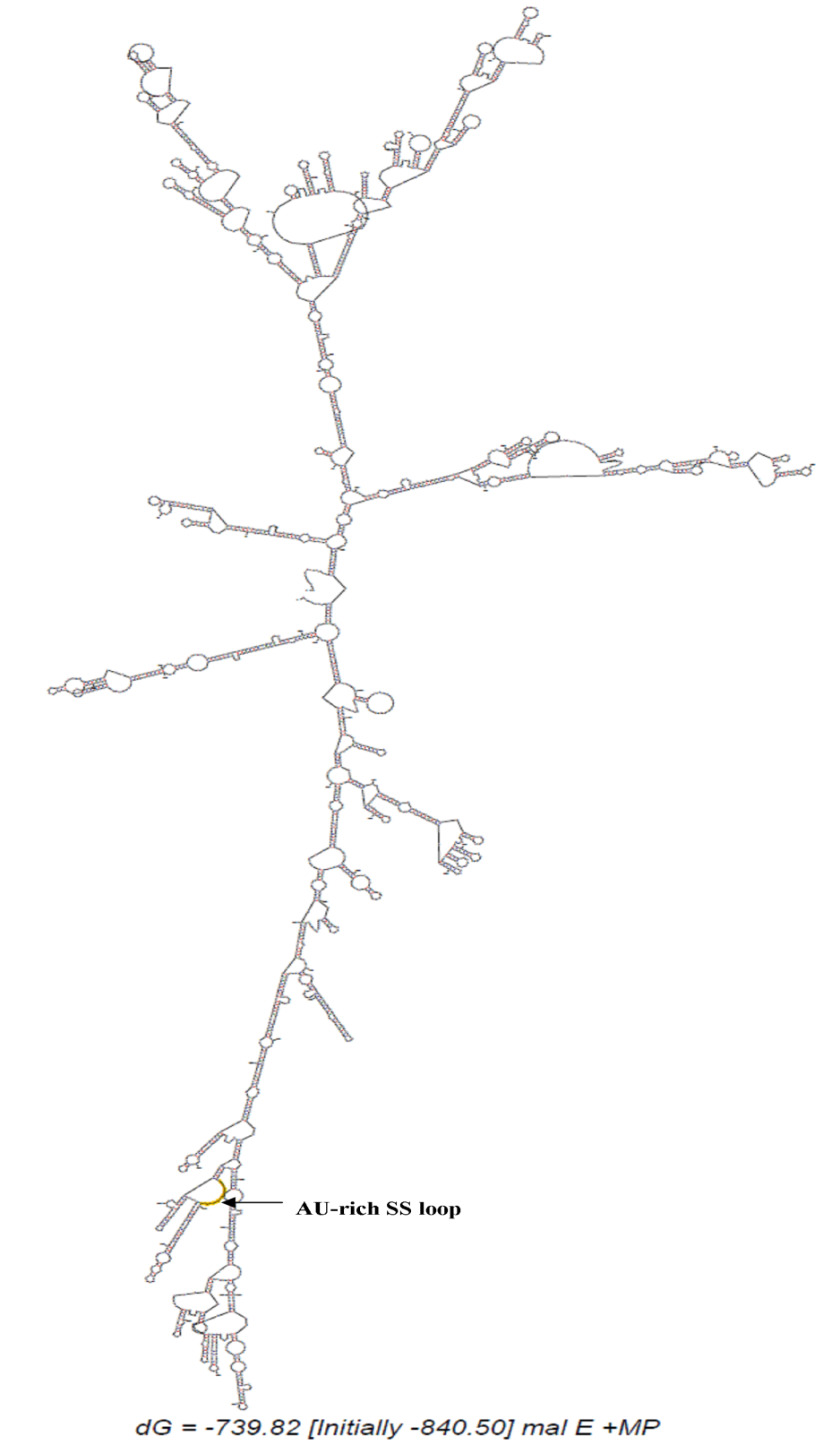
**

**Supplementary Fig. 1**: Predicted secondary structure of chimeric (malE+ACLSV MP) RNA showing the single stranded (SS) AU rich loop spanning the region between nucleotides 2103-2119 (highlighted with yellow color) which is a potential site for cleavage by RNase E. The secondary structure was predicted at mFOLD web server (<http://unafold.rna.albany.edu/?q=mfold/RNA-Folding-Form>).


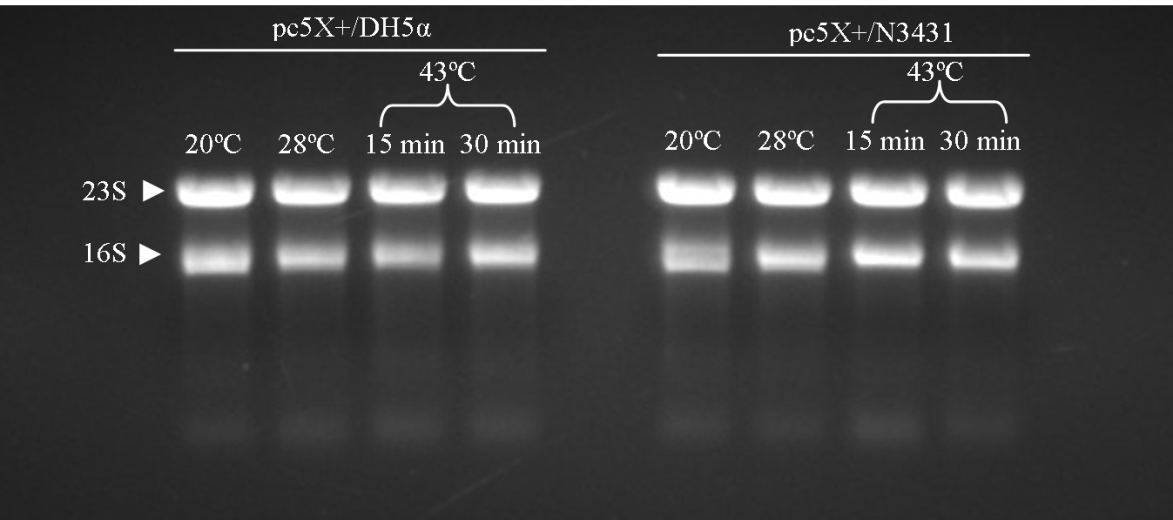


**Supplementary Fig. 2.** EtBr stained total RNA image showing 23S and 16S bands from pc5X+/DH5α (left panel) and pc5X+/N3431 (right panel). For loading control, the relative intensities of 23S bands were calculated using Gene Tools software Version 4.02 (SynGene, Cambridge, England). The relative intensities of each band are presented as a separate excel file (Supplementary file 2).


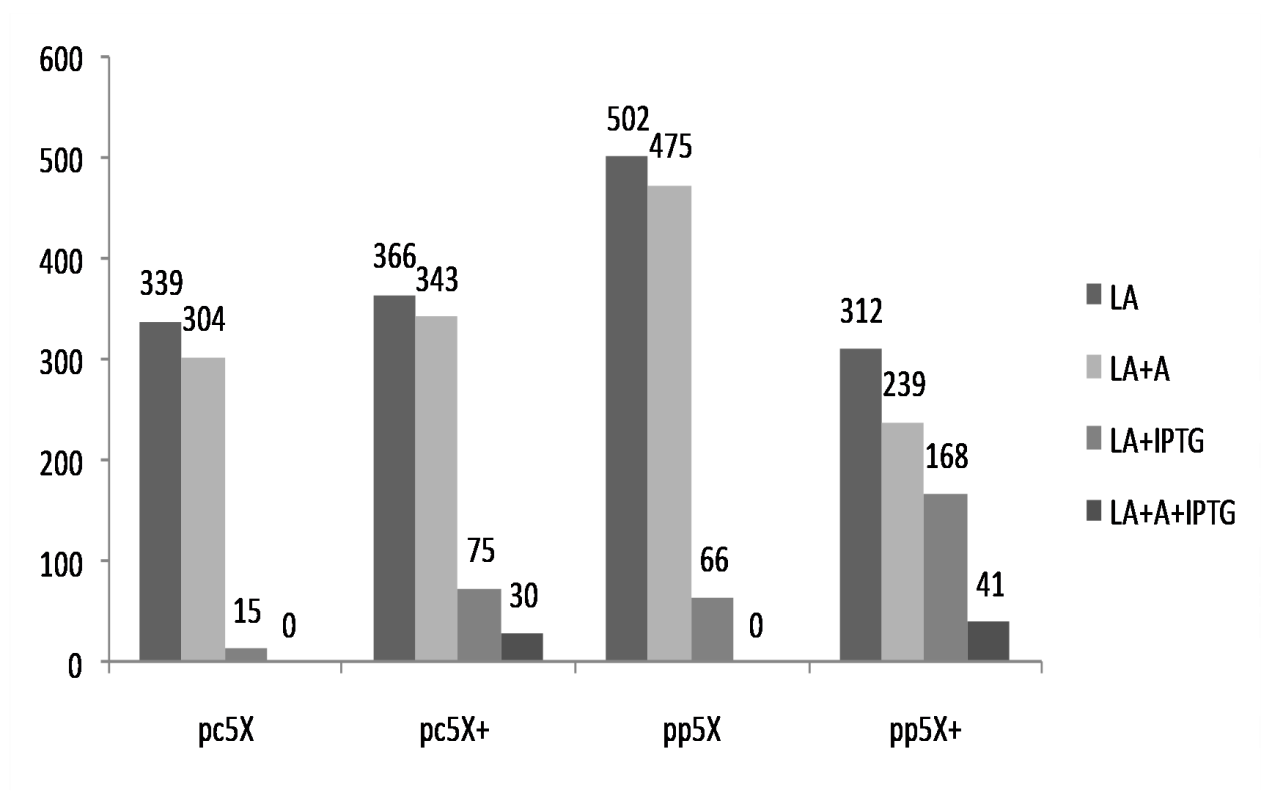


**Supplementary Fig. 3:** Graphical representation of plasmid stability test carried out using pMAL vector controls (pc5X and pp5X) and chimeric plasmids (pc5X+ and pp5X+). Vector controls and chimeric plasmids were transformed into NEB express cells. Single positive colony from each transformation was grown in luria broth till O.D_600_ reached 0.5. Equal numbers of cells from each culture were then spread on following plates (1) Luria agar (LA), (2) Luria agar containing 100 µg/ml ampicillin (LA+A), (3) Luria agar containing 0.3 mM IPTG (LA+IPTG), (4) Luria agar containing ampicillin and IPTG at concentrations mentioned above. The number of colonies represented on each bar is the average of three independent experiments.

**Supplementary Table. 1.** Relative intensities of 23S bands used as internal loading control in northern blot experiment (Fig.5). The values in red are the relative intensity values (as compared to band 1 of track 1) for 23S ribosomal RNA band.

| **Track 1** |  |  |  |  |  |
| --- | --- | --- | --- | --- | --- |
| **Number** | **Mol. weight** | **Height** | **Raw vol.** | **Quantity** |  |
| 1 | 0 | 176.7326 | 89183.55 | 1 | 23S |
| 2 | 0 | 139.0175 | 75973.97 | 0.851883 |  |
| 3 | 0 | 9.326297 | 6104.651 | 0.06845 |  |
| **Track 2** |  |  |  |  |  |
| **Number** | **Mol. weight** | **Height** | **Raw vol.** | **Quantity** |  |
| 1 | 0 | 175.5925 | 84167.58 | 0.943757 | 23S |
| 2 | 0 | 128.5675 | 65389.59 | 0.733202 |  |
| 3 | 0 | 11.9787 | 9357.682 | 0.104926 |  |
| **Track 3** |  |  |  |  |  |
| **Number** | **Mol. weight** | **Height** | **Raw vol.** | **Quantity** |  |
| 1 | 0 | 170.1005 | 83133.08 | 0.932157 | 23S |
| 2 | 0 | 110.7617 | 59968.26 | 0.672414 |  |
| 3 | 0 | 12.0326 | 12135.06 | 0.136068 |  |
| **Track 4** |  |  |  |  |  |
| **Number** | **Mol. weight** | **Height** | **Raw vol.** | **Quantity** |  |
| 1 | 0 | 170.2576 | 85245.94 | 0.955848 | 23S |
| 2 | 0 | 137.3787 | 70606.5 | 0.791699 |  |
| 3 | 0 | 11.21751 | 7358.701 | 0.082512 |  |
| **Track 5** |  |  |  |  |  |
| **Number** | **Mol. weight** | **Height** | **Raw vol.** | **Quantity** |  |
| **Track 6** |  |  |  |  |  |
| **Number** | **Mol. weight** | **Height** | **Raw vol.** | **Quantity** |  |
| 1 | 0 | 172.682 | 88610.02 | 0.993569 | 23S |
| 2 | 0 | 112.307 | 69322.79 | 0.777305 |  |
| 3 | 0 | 9.319257 | 8688.218 | 0.09742 |  |
| **Track 7** |  |  |  |  |  |
| **Number** | **Mol. weight** | **Height** | **Raw vol.** | **Quantity** |  |
| 1 | 0 | 174.4156 | 83779.37 | 0.939404 | 23S |
| 2 | 0 | 142.8117 | 71103.24 | 0.797269 |  |
| 3 | 0 | 8.338849 | 8160.972 | 0.091508 |  |
| **Track 8** |  |  |  |  |  |
| **Number** | **Mol. weight** | **Height** | **Raw vol.** | **Quantity** |  |
| 1 | 0 | 177.4326 | 90776.8 | 1.017865 | 23S |
| 2 | 0 | 159.9537 | 74798.58 | 0.838704 |  |
| 3 | 0 | 15.47548 | 14238.85 | 0.159658 |  |
| **Track 9** |  |  |  |  |  |
| **Number** | **Mol. weight** | **Height** | **Raw vol.** | **Quantity** |  |
| 1 | 0 | 176.3175 | 88142.94 | 0.988332 |  |
| 2 | 0 | 146.9925 | 70290.08 | 0.788151 |  |
| 3 | 0 | 9.203705 | 9828.41 | 0.110204 |  |
